# Risk of Venous Thromboembolism in Patients with Cancer: A Systematic Review and Meta-Analysis

**DOI:** 10.1371/journal.pmed.1001275

**Published:** 2012-07-31

**Authors:** Freesia Horsted, Joe West, Matthew J. Grainge

**Affiliations:** Division of Epidemiology and Public Health, University of Nottingham, Nottingham, United Kingdom; Leiden University Medical Center, Netherlands

## Abstract

**Background:**

People with cancer are known to be at increased risk of venous thromboembolism (VTE), and this risk is believed to vary according to cancer type, stage of disease, and treatment modality. Our purpose was to summarise the existing literature to determine precisely and accurately the absolute risk of VTE in cancer patients, stratified by malignancy site and background risk of VTE.

**Methods and Findings:**

We searched the Medline and Embase databases from 1 January 1966 to 14 July 2011 to identify cohort studies comprising people diagnosed with one of eight specified cancer types or where participants were judged to be representative of all people with cancer. For each included study, the number of patients who developed clinically apparent VTE, and the total person-years of follow-up were extracted. Incidence rates of VTE were pooled across studies using the generic inverse variance method. In total, data from 38 individual studies were included. Among average-risk patients, the overall risk of VTE was estimated to be 13 per 1,000 person-years (95% CI, 7 to 23), with the highest risk among patients with cancers of the pancreas, brain, and lung. Among patients judged to be at high risk (due to metastatic disease or receipt of high-risk treatments), the risk of VTE was 68 per 1,000 person-years (95% CI, 48 to 96), with the highest risk among patients with brain cancer (200 per 1,000 person-years; 95% CI, 162 to 247). Our results need to be considered in light of high levels of heterogeneity, which exist due to differences in study population, outcome definition, and average duration of follow-up between studies.

**Conclusions:**

VTE occurs in greater than 1% of cancer patients each year, but this varies widely by cancer type and time since diagnosis. The absolute VTE risks obtained from this review can aid in clinical decision-making about which people with cancer should receive anticoagulant prophylaxis and at what times.

*Please see later in the article for the Editors' Summary.*

## Abstract 2

Venous thromboembolism in patients with cancer is common, but precise incidence rates in different cancers are not known, making it difficult to target prevention strategies. This study summarizes the existing literature to determine the risk of venous thromboembolism in high- and average-risk groups of patients with different cancers.

## Editors' Summary

**Background:**

A venous thrombosis is the medical term for a blood clot that forms in a vein, often completely blocking the vessel. The most common type is a deep vein thrombosis of the lower leg, which apart from causing pain and immobility, can break off (embolize), flow through the blood stream back to the heart, get caught in one of the blood vessels supplying the lungs, and cause a life-threatening pulmonary embolism. The term venous thromboembolism (VTE) refers to both a deep venous thrombosis and a pulmonary embolism and is a common cause of death, responsible for at least 300,000 deaths a year in the United States alone. There are many risk factors for developing a VTE, including age, immobility, certain medications, and some conditions, such as cancer: an estimated 20% of deaths from VTE occur among patients with cancer, and importantly, cancer patients with VTE have a much higher risk of death than those who do not have a VTE. The increased risk of developing a VTE is due to the treatments and surgery involved in the management of cancer, in addition to the risks associated with the condition itself.

**Why Was This Study Done?:**

Previous studies have suggested that certain types of cancer, such as brain and pancreatic cancer, are associated with an increased risk of developing a VTE, but to date, clinical guidelines recommend preventative treatment of VTE only for cancer patients during hospital admissions for medical treatment and surgery, not for those patients receiving outpatient care. In this study, the researchers systematically reviewed the available published evidence to quantify the risks of developing a VTE in patients with cancer according to the type of cancer, and to determine whether certain patient groups are at particularly high risk of developing a VTE.

**What Did the Researchers Do and Find?:**

The researchers used a comprehensive keyword search of two medical literature databases to identify relevant studies published between 1966 and 2011. Then they examined these studies according to certain criteria, such as the type of study and the type of cancer: the researchers were specifically looking for cohort studies of adult patients with one of eight cancer types—breast, lung, colorectal, prostate, brain, bone, pancreatic, and hematologic (including all leukemias, lymphomas, and multiple myeloma). The selected studies also had to include follow-up of more than 30 days and VTE outcomes. Then the researchers categorized selected studies according to the risk of developing a VTE—the researchers judged high-risk patients to be those with metastatic disease or receiving certain types of high-risk treatments, and judged average-risk patients to be representative of all patients with a cancer diagnosis. The researchers then pooled all the data from these studies and did a separate statistical analysis for high and average risk and for each cancer type.

Using these methods, the researchers identified 7,274 potentially relevant articles, of which 46 reports from 38 individual cohorts met the criteria to be included in their review. Of the 38 cohorts, the researchers categorized 31 as high risk and seven as average risk. In the pooled analysis the researchers found that among average-risk patients, the overall risk of VTE was 13 per 1,000 person-years, with the highest risk among patients with cancers of the pancreas, brain, and lung. Among patients judged to be at high risk, the researchers found that the risk of VTE was 68 per 1,000 person-years, with the highest risk among patients with brain cancer (200 per 1,000 person-years).

**What Do These Findings Mean?:**

These findings suggest that the annual incidence rate of VTE in patients with cancer is between 0.5% and 20%, depending on the cancer type, background risk, and time since diagnosis. Cancers of the brain and pancreas have the highest risk of VTE for both high- and average-risk patient groups. Based on these more accurate data on the risks of VTE in different groups of cancer patients, future updates of clinical guidelines can now include more information about categories of risk to help guide clinicians when they make decisions about which patients should receive preventative treatment for VTE and when they should receive such treatment.

**Additional Information:**

Please access these websites via the online version of this summary at http://dx.doi.org/10. 1371/journal.pmed.1001275.

- Wikipedia gives more information about VTE (note that Wikipedia is a free online encyclopedia that anyone can edit)
- Information about VTE for patients and health professionals is available from the American Cancer Society, the US National Cancer Institute, and the UK-based thrombosis charity Lifeblood

## Introduction

Venous thromboembolism (VTE), which includes deep venous thrombosis and pulmonary embolism, is the third most common circulatory disorder in Western populations, and in the United States alone is responsible for an estimated 300,000 deaths annually [1]. It has been estimated that 20% of these deaths occur among patients with cancer [2], and that the risk of death is more than three times higher for cancer patients with VTE than for those without VTE [3]. Whilst the overall incidence of cancer has declined over the last two decades, improvements in cancer survival and changes in the age structure of the population mean there are now more people in the US living with this illness than before [4],[5]. Furthermore, those cancer treatments such as surgery and chemotherapy to which this improved survival over time has been attributed are themselves believed to directly increase VTE risk [6],[7]. Further improvements in cancer survival could therefore be obtained through more careful targeting of VTE prophylaxis at both the highest risk patients and at the most appropriate times within these patients' disease course [8]. Existing clinical guidelines recommend primary VTE prophylaxis for cancer patients during medical and surgical hospitalisations (where not contra-indicated) [9]–[12], with recent updates to two sets of guidelines suggesting that some outpatient chemotherapy patients could also benefit [11],[12]. Further research is clearly required before more intricate risk stratification can be introduced. Such risk stratification, however, is difficult in the absence of clear data on the absolute VTE risk of patients with different cancers over a specifically defined period of time and how risk varies according to factors such as stage of disease and treatment modality.

Data from hospital discharge episodes indicate that VTE is most likely to occur in patients with brain, pancreatic, and haematological tumours, when data are adjusted for the prevalence of these cancer types [13]–[15]. Equivalent evidence from cohort studies is difficult to interpret because of the absence of previous efforts to systematically evaluate research data from a diversity of sources characterising the incidence of VTE among people with different cancers. The aim of this systematic review was to use published literature to determine the absolute and relative risk of symptomatic VTE in cancer patients, stratified by cancer type and whether patients were considered to be at particularly high risk of VTE.

## Methods

### Data Sources and Searches

This review was carried out and reported in accordance with the PRISMA guidelines for systematic reviews and meta-analyses (Text S1) [16]. A comprehensive search of the Medline (OVID) and Embase databases from 1 January 1966 to 14 July 2011 was carried out to identify published studies that provided a quantitative estimate of the incidence of VTE in cancer patients, along with control groups where available (Texts S2 and S3). Reference lists of appropriate review articles and of the original retrieved studies were searched to identify studies missed by the database searches. No original protocol for the review was produced.

### Study Selection

Two authors (F. H. and M. J.G.) reviewed titles, abstracts, and full text articles, with any discrepancies about study inclusion resolved by discussion among all three authors. Inclusion and exclusion of papers was based on the following criteria.

#### Study design

We included reports from prospective or retrospective cohort studies. All data from randomised controlled trial participants were excluded, as these patients are frequently recruited following strict inclusion and exclusion criteria so are liable to be unrepresentative of the underlying population of interest. For instance, very ill patients are usually less likely to be recruited into trials [17].

#### Participants

Included studies involved adult patients diagnosed with one of the following eight cancer types: breast, lung, colorectal, prostate, brain, bone, pancreatic, or haematologic (including all leukaemias, lymphomas, and multiple myeloma). The first four cancer types were chosen because they are the most prevalent malignancy types in the United Kingdom [18]. The other types were selected because previous research suggests that these types are associated with the highest risk of VTE. Studies that presented data for all cancer patients (or where the types under investigation accounted for more than 75% of all cancers based on UK data [18]) were used to provide our estimate of the risk of VTE in all cancer patients (averaged across cancer types). Studies focussing predominantly on patients fitted with indwelling catheters were not considered. No restrictions were made on the basis of nationality or on the stage or grade of malignancy. Studies comprising patients with VTE at baseline were excluded, unless data were presented separately for patients with and without a previous VTE. Before the review commenced a decision was made to exclude any study with fewer than 20 participants, as it was unlikely that these would produce a sufficient number of people developing VTE to contribute meaningful information.

#### Follow-up

To enable the total person-years of observation to be calculated, we included data from reports that specified one or more of the following: (i) total person-time of follow-up, (ii) sample size and mean (or median) follow-up per patient, or (iii) sample size and cumulative incidence rate. We excluded studies where the average duration of follow-up was less than 30 d, hence studies containing only in hospital follow-up following a cancer-related procedure did not form part of this review.

#### Outcomes

We included reports that contained information on the number of patients in the study who developed a primary, clinically apparent VTE over the course of the study. Usually this would be a conjugate outcome comprising deep vein thrombosis and pulmonary embolism. Where only one of these events was considered, these studies were included but this was clearly stated. Any multiple or recurrent events were excluded.

### Data Extraction and Quality Assessment

Data were extracted independently by two authors (F. H. and M. J. G.). When multiple publications were available from a single cohort, we extracted data from the paper that provided information on the greatest total duration of follow-up for each cancer type (and overall cancer) in order to maximise information. We did not formally assess the quality of the studies included in the review. This was because available assessment tools such as the Newcastle-Ottawa scale for cohort studies are based on criteria including “the selection of control subjects” and “degree of adjustment for confounders” that cannot easily be adapted to situations where the primary reason for reviewing the paper is to extract data on incidence. Specific methodological issues—such as how factors including duration of follow-up and ascertainment of VTE in the source studies could impact the overall findings from this review—were considered carefully.

For each study the number of patients who developed VTE and the total person-years of follow-up were extracted from the study report. Where the total person-years of follow-up was not explicitly stated, this was obtained by multiplying the mean follow-up per patient by the number of participants (using the median as an approximation if only this was available). If only the number of patients and cumulative incidence were provided (defined as the number of people who developed VTE over a specified time period following diagnosis/treatment, ignoring the potential for censoring due to death), then total person-years of follow-up could be calculated as long as either the median survival or the percentage of the sample alive at the end of the period under study was specified. In this instance, the total person-years of follow-up was estimated assuming an exponential survival function.

Where study reports specified the number of people (and VTE events) with each cancer type but where average follow-up was stated only for the cohort as a whole, we felt it was not reasonable to assume equal follow-up for each cancer type, given the widely different prognosis (i.e., survival) for different cancers. Two exceptions were made to this. In one study, the duration of follow-up for all patients was short (2.4 mo), such that there would be little opportunity for differential prognosis to impact on average follow-up times [19]. In the second study, participants were followed up for a maximum of 2 y, and person-years of follow-up for each cancer type could be estimated using available data on the probability of surviving to 2 y for the specified cancer type and assuming exponential survival [20]. When analyses were repeated with the exclusion of these two studies, the results did not change appreciably.

### Categorisation of Studies as Average or High Risk

We stratified papers by whether study participants were average (population-based) risk or high risk based on their underlying risk of VTE. We defined average-risk populations as those where we judged that the participants were representative of all patients with the cancer type under investigation (or overall cancer). High-risk populations were those where all or the majority of participants either had high-grade or metastatic disease, or underwent procedures for treating the underlying malignancy that are believed to increase thromboembolic risk, including surgery, radiotherapy, and chemotherapy [6],[7]. Decisions on whether to categorise study populations as average or high risk were made jointly by all three authors.

### Data Synthesis and Analysis

All analyses were carried out using Stata v. 11. For each study, the natural logarithm of the incidence rate (number new cases/1,000 person-years) was estimated, along with the standard error (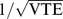 events) [21]. These were then pooled for each cancer type assuming random effects using the generic inverse variance method. This method considers the inverse of the variance of the effect estimate, i.e., 1/(Standard Error)^2^, as the weight given to each study, so studies with more VTE events are given greater weight than studies with fewer events. Heterogeneity, the variation between study results, was assessed using the *I* ^2^ statistic [22]. Usually, where the degree of heterogeneity is large (*I* ^2^>75%), subgroup analyses are encouraged to explore the between-study heterogeneity. However, in the present review we felt the overall number of studies in each analysis was too small for such an analysis to be meaningful.

We performed separate analyses for high- and average-risk populations, as described above, as well as for each cancer type. Given the somewhat arbitrary judgement involved in categorising study populations as either high or average risk, we performed an additional analysis restricting the former group to study populations that were categorised as high risk on the basis of having received cancer treatments at baseline. Statistical methods for pooling incidence rate ratios were not required here because only one study meeting the inclusion criteria contained a control group for which both the number of VTE events and person-years of follow-up were explicitly stated.

## Results

### Selection of Studies

**Figure 1:**
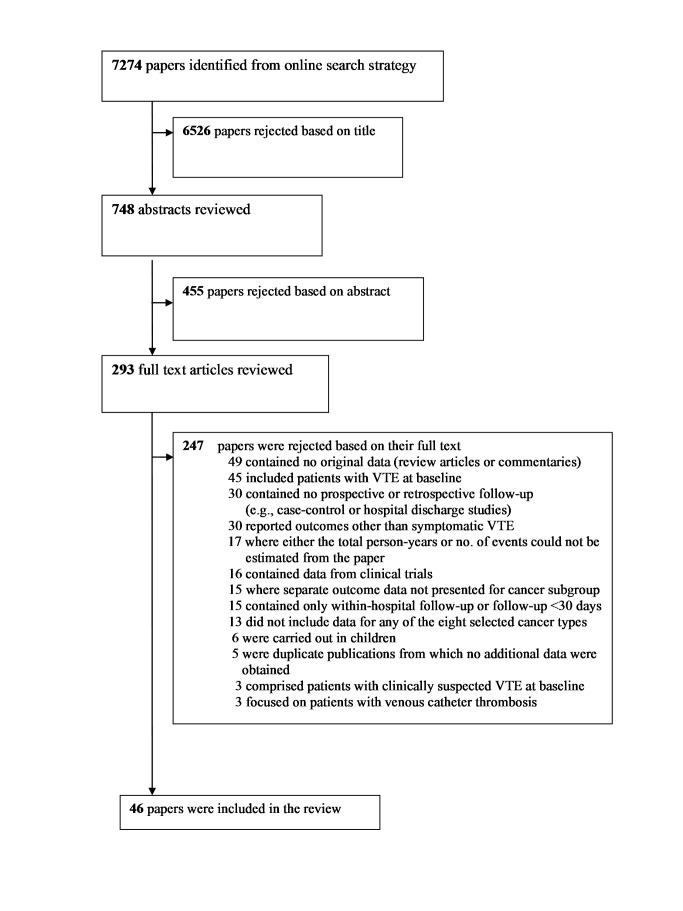
Flow diagram for study selection.

A total of 7,274 articles were identified via our search strategy, and the full text was retrieved for 293 articles. Of these, 46 reports from 38 individual cohorts (studies) were included in the review [19],[20],[23]–[66] (Figure 1). There were six separate papers from a single cohort from California (one providing incidence rates of VTE for 15 separate cancer types [33], and five individual reports focussing on cancers of the lung, breast, bowel, brain, and leukaemia [24],[32],[34],[45],[56]). In a separate study of outpatient chemotherapy patients [19], a subsequent publication became available that provided information on a greater number of patients with respect to overall cancer risk [35]. The original paper from the Austrian Cancer and Thrombosis Study (CATS) [20] was used to extract data for overall and haematological malignancies, whilst data on larger numbers of people with specific solid tumours were available from two subsequent publications [41],[62].

Among the papers excluded at the full text stage, 17 were from cohorts that were otherwise eligible, except for the absence of sufficient information in the paper to be able to estimate both the total person-years of follow-up and number of VTE events [67]–[83]. All included studies were identified from the search terms listed in Texts S2 and S3. A careful investigation of two recent review articles on this topic did not result in the identification of any additional studies that met our review criteria [8],[84].

### Overview of Included Studies

Details of included studies are summarised in Table S1. Of the 38 included studies, 20 were from Europe, 14 from North America (US and Canada), one contained participants from both the US and Europe, and three studies were conducted in Asia. One study using the SEER-Medicare database was restricted to people aged 65 y old and over [39]; the average age (mean or median) of participants at baseline for all other studies ranged from 47 to 68 y.

Of the 38 cohorts, 31 were categorised as high risk and seven were categorised as average risk because they were judged to be representative of all patients with a cancer diagnosis [30],[33],[36],[40],[57],[61],[64]. Studies were classed as high risk when follow-up commenced following outpatient chemotherapy (*n* = 9), surgery (*n* = 8), inpatient hospitalisation (not specifically for surgery, *n* = 2), or a receipt of a mixture of treatment types (*n* = 7), or because either all or the majority of patients had advanced or metastatic cancer at baseline (*n* = 5). Prophylaxis was administered to either some (>20%) or all of the participants in 11 studies; with this intervention taking the form of either anticoagulant prophylaxis (with or without mechanical methods) [23],[31],[47],[53],[55],[58],[59], mechanical prophylaxis only [25],[27], aspirin [42], or unspecified prophylaxis [28]. In a further six studies it was stated explicitly that no patients [20],[26],[37],[38],[43],[44] were receiving anticoagulant prophylaxis, and in two further studies [29],[66] there was a small number (<5%) receiving warfarin or heparin. In all other reports this information was either not known or not reported.

### Overall Risk of VTE

**Figure 2:**
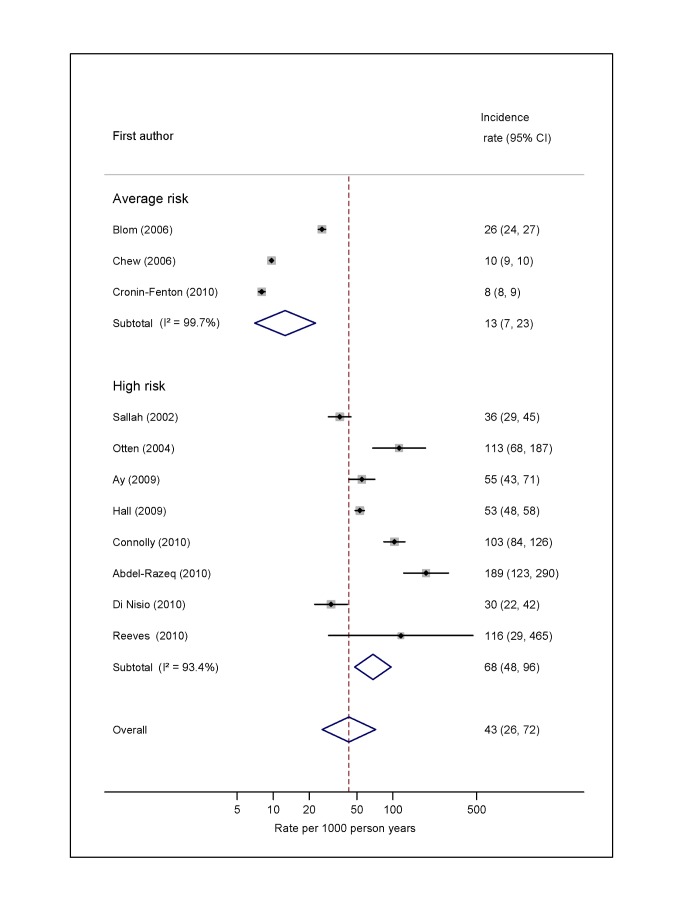
Pooled incidence of venous thromboembolism for overall cancer. Natural logarithms of the incidence rate are presented on the *x*-axis. Black diamonds indicate the point estimate (VTE incidence) for each individual study. Horizontal lines indicate the 95% confidence interval surrounding this estimate. Open blue diamonds describe both the point estimate (centre of the diamond) for the pooled VTE incidence for average-risk, high-risk, and all studies (average- and high-risk together), and the 95% confidence interval for this pooled estimate (width of the diamond). Blom (2006) indicates data from [30].

For average-risk studies, incidence rates of VTE ranged from 8 per 1,000 person-years over an average of 27 mo in Denmark [36] to 26 per 1,000 person-years in the 6 mo following diagnosis in the Netherlands [30] (pooled incidence rate 12.6 per 1,000 person-years; 95% CI, 7.0 to 22.6; heterogeneity *I* ^2^>99%) (Figure 2; Table S2). The pooled incidence rate from eight studies combining data from high-risk samples was 68.0 per 1,000 person-years (95% CI, 48.0 to 96.4; heterogeneity *I* ^2^ = 93.4%), with average follow-up durations ranging from 1 mo to 26 mo.

### Risk of VTE by Cancer Type

**Figure 3:**
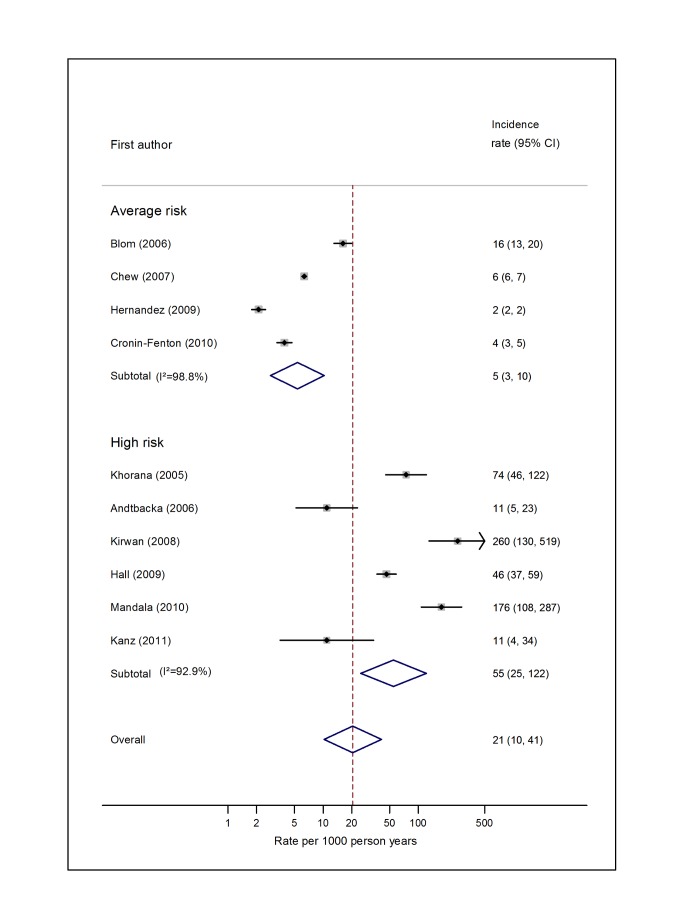
Pooled incidence of venous thromboembolism for breast cancer. Natural logarithms of the incidence rate are presented on the *x*-axis. Symbols as in Figure 2. Blom (2006) indicates data from [30].

Pooled estimates for all eight cancer types are summarised in Figures 3–10 and Tables S3, S4, S5, S6, S7, S8, S9, S10. Cancers of the pancreas (59 per 1,000 person-years) and brain (48 per 1,000 person-years) were associated with the greatest and second greatest risk of VTE among average-risk patients, whilst their relative importance was reversed among high-risk groups (brain, 200 per 1,000 person-years; pancreas, 155 per 1,000 person-years). Prostate cancer was found to be associated with a low risk of VTE in both average- (8 per 1,000 person-years) and high- (19 per 1,000 person-years) risk studies (Figure 6; Table S6); breast cancer was associated with the lowest risk of VTE among average-risk groups (5 per 1,000 person-years), but among those at high risk of VTE, this rose to 55 per 1,000 person-years (Figure 3; Table S3). Patients with colorectal, lung, and haematological malignancies had risks of a similar magnitude to the rate among all cancer patients. Only two studies from average-risk populations provided data on bone cancer, and these provided strikingly different estimates of the VTE risks in these patients. *I* ^2^ values ranged from 74.4% to 98.8% when pooling average-risk studies and from 0.0% to 92.9% when pooling high-risk studies.

**Figure 4 pmed-1001275-g004:**
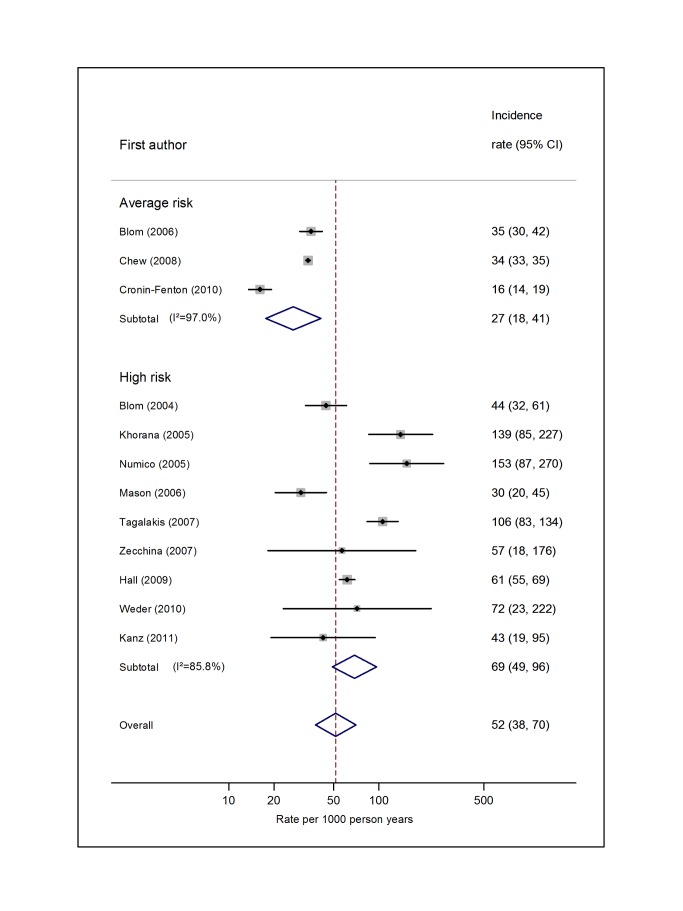
Pooled incidence of venous thromboembolism for lung cancer. Natural logarithms of the incidence rate are presented on the *x*-axis. Symbols as in Figure 2. Blom (2006) indicates data from [30].

**Figure 5 pmed-1001275-g005:**
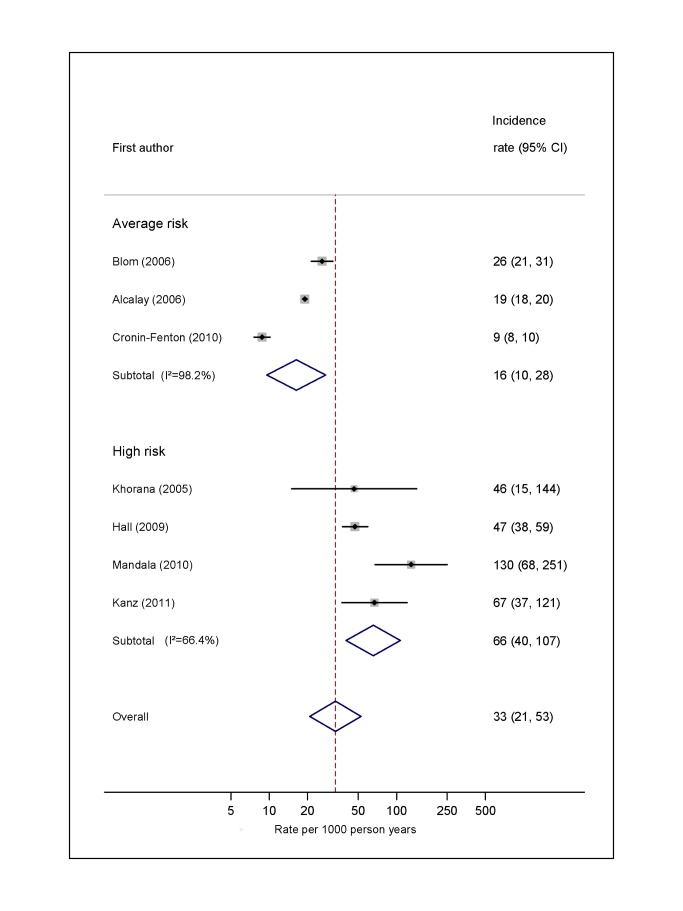
Pooled incidence of venous thromboembolism for colorectal cancer. Natural logarithms of the incidence rate are presented on the *x*-axis. Symbols as in Figure 2. Blom (2006) indicates data from [30].

**Figure 6 pmed-1001275-g006:**
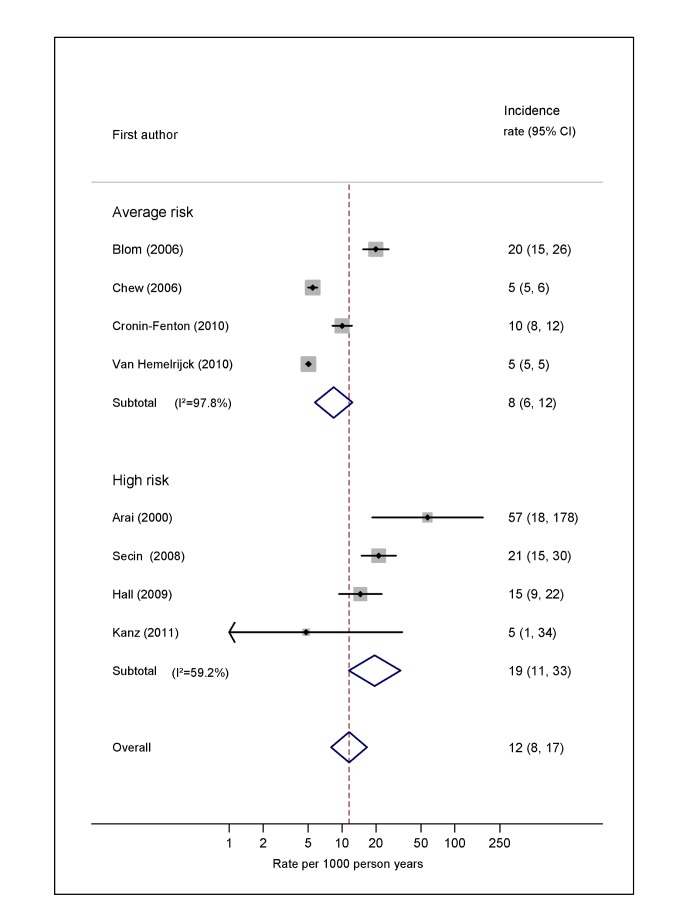
Pooled incidence of venous thromboembolism for prostate cancer. Natural logarithms of the incidence rate are presented on the *x*-axis. Symbols as in Figure 2. Blom (2006) indicates data from [30].

**Figure 7 pmed-1001275-g007:**
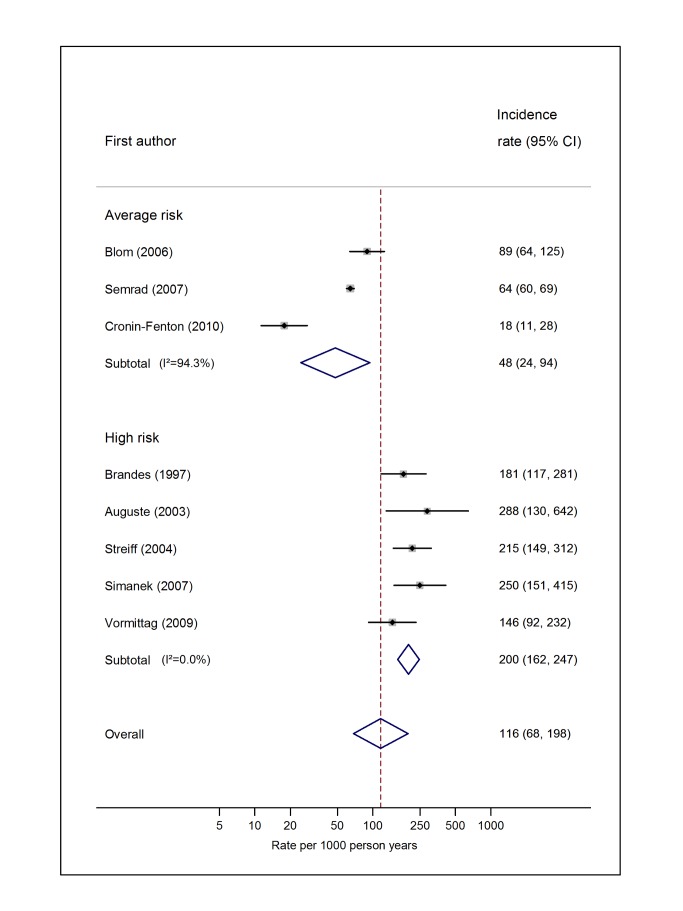
Pooled incidence of venous thromboembolism for brain cancer. Natural logarithms of the incidence rate are presented on the *x*-axis. Symbols as in Figure 2. Blom (2006) indicates data from [30].

**Figure 8 pmed-1001275-g008:**
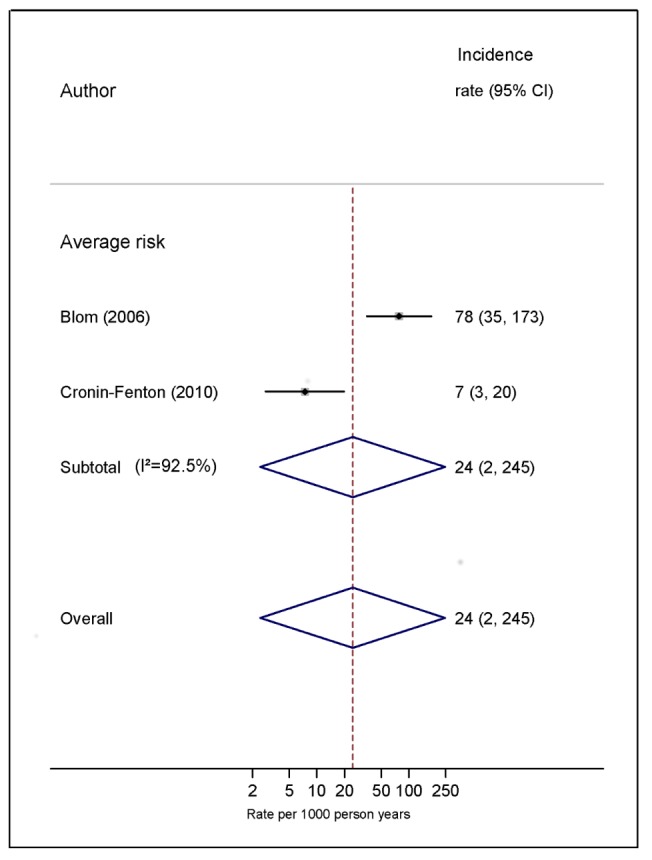
Pooled incidence of venous thromboembolism for bone cancer. Natural logarithms of the incidence rate are presented on the *x*-axis. Symbols as in Figure 2. Blom (2006) indicates data from [30].

**Figure 9 pmed-1001275-g009:**
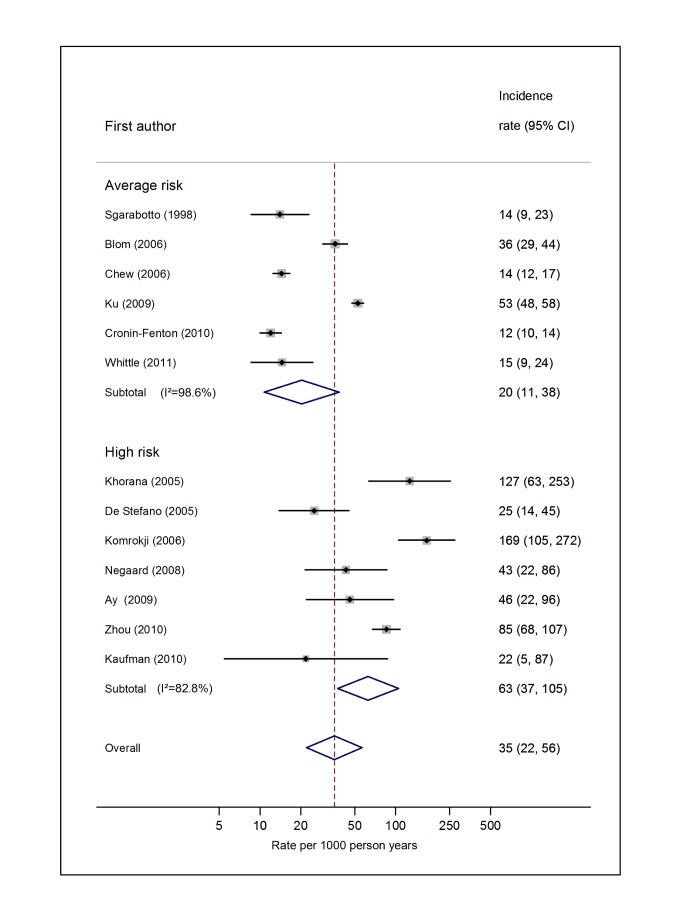
Pooled incidence of venous thromboembolism for haematological cancer. Natural logarithms of the incidence rate are presented on the *x*-axis. The studies reported by Chew et al. [33] (non-Hodgkin lymphoma) and Ku et al. [45] (leukaemia) were both from the California Cancer Registry cohort. However, because they included different subsets of patients and were conducted over different time intervals (Chew, 1993–1995, and Ku, 1993–1999), a decision was made to pool these as separate studies. Symbols as in Figure 2. Blom (2006) indicates data from [30].

**Figure 10:**
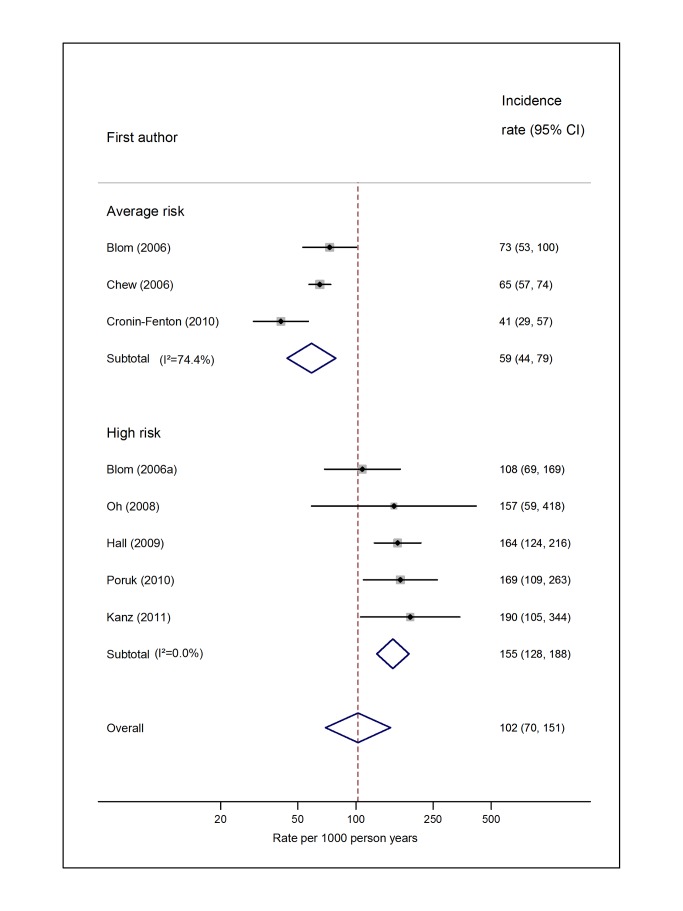
Pooled incidence of venous thromboembolism for pancreatic cancer. Natural logarithms of the incidence rate are presented on the *x*-axis. Blom (2006) indicates data from [30]. Blom (2006a) indicates data from [29].

### Risk of VTE when Follow-Up Commenced at Cancer Diagnosis

Figure S1 summarises the pooled estimates of VTE for each cancer type for all studies where follow-up commenced at the time of cancer diagnosis regardless of whether the study was classed as average or high risk in the previous analysis (*n* = 14 cohorts). Whilst the relative importance of the cancer sites is similar to that in the average-risk analysis, absolute risks of VTE are usually higher in this instance (ranging from 7.7 per 1,000 person-years for breast cancer to 110.1 per 1,000 person-years for pancreatic cancer).

### Risk of VTE following High-Risk Treatments

When the analysis of high-risk groups was restricted to studies of patients receiving high-risk treatments at baseline (*n* = 26), the risk of VTE among all cancer patients increased slightly to 72.7 per 1,000 person-years (95% CI, 44.2 to 119.5). For several individual cancer types there were also small increases in the pooled risk after making this restriction, the largest of which occurred among people with colorectal cancer (81.0 per 1,000 person-years; 95% CI, 46.7 to 141.2; heterogeneity *I* ^2^ = 40.2%, *n* = 3 studies) and brain cancer (217.1 per 1,000 person-years; 95% CI, 171.5 to 274.9; heterogeneity *I* ^2^ = 0.0%, *n* = 4 studies) (data not shown). Only for lung cancer was the pooled risk reduced slightly after making this restriction (67.5 per 1,000 person-years; 95% CI, 39.6 to 115.1; heterogeneity *I* ^2^ = 83.9, *n* = 7 studies).

Further restriction of brain cancer studies to only those where patients received anticoagulants as prophylaxis around the time of surgery (*n* = 3 studies) had little effect on the pooled estimate (211.3 per 1,000 person-years; 95% CI, 165.1 to 270.5; heterogeneity *I* ^2^ = 0.0%). There were insufficient numbers of studies comprising patients receiving anticoagulants to perform an equivalent analysis for other cancer types.

### Relative Risk of VTE Compared with the General Population

**Figure 11:**
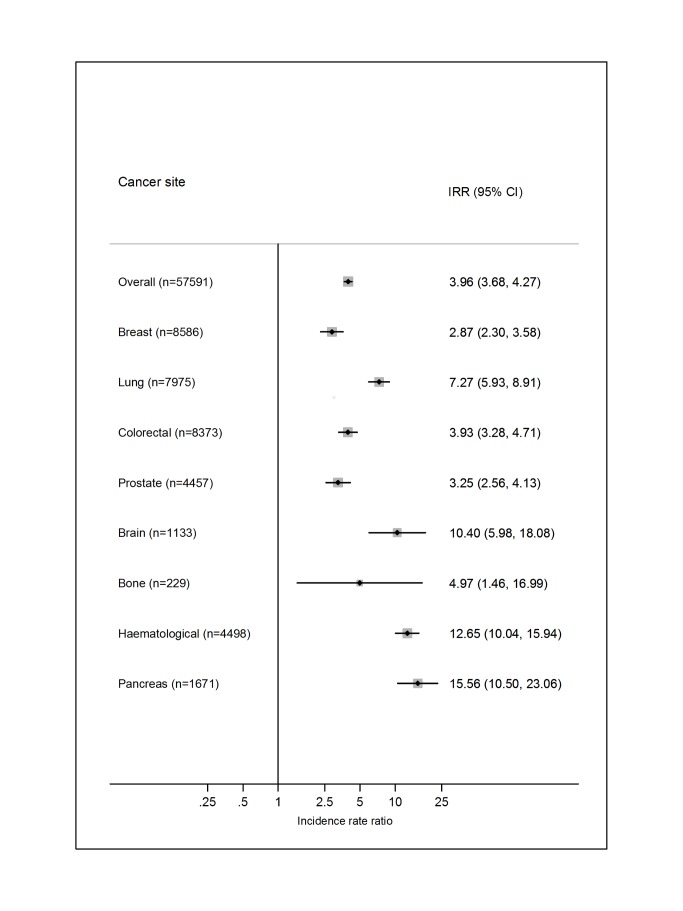
Relative risks of venous thromboembolism in cancer patients compared with in the general population. Results for selected cancer types obtained from Cronin-Fenton et al. [36]. IRR, incidence rate ratio.

Just one of the included studies [36] provided a direct comparison with the risk of VTE in the general population, whereby five controls were matched to each cancer patient specifically on birth, sex, and region (Figure 11). Overall, the risk of VTE was increased 4-fold in cancer patients (incidence rate ratio = 3.96; 95% CI, 3.68 to 4.27). The relative importance of individual cancer types reflected those for the absolute VTE risks, except for a high incidence rate ratio for haematological cancer, reflecting the lower rate in the mainly younger matched controls linked to people with this type of cancer.

## Discussion

In a pooled analysis of data from 38 study populations, we estimated the annual incidence rate of VTE to be between 0.5% and 20% depending on the cancer type and background risk. Cancers of the brain and pancreas were associated with the highest risk of VTE both within study populations classed as high risk and within those classed as average risk. Current guidelines recommend VTE prophylaxis for hospitalised cancer patients and for selected high-risk ambulatory patients who are receiving systemic chemotherapy [9]–[12]. By providing more accurate data on the absolute risks of VTE in different groups and weighing these against the bleeding risks entailed with such therapy, future updates of these guidelines can incorporate more careful risk stratification to highlight which patients should receive prophylaxis.

Our systematic review and meta-analysis has several strengths. First, this is, to our knowledge, the first occasion that a review of the literature on this important topic has attempted to quantify precisely the likelihood of this potentially devastating complication, taking account of person-time denominators starting from the time of cancer diagnosis or treatment. Second, we used two databases to search the available literature, with search terms refined to ensure maximum sensitivity. This was supplemented by searches of reference lists and relevant review articles to ensure no papers were missed. Third, our separation of studies into high- and average-risk patient groups achieved two important purposes: it helped alleviate in part the problem of heterogeneity described below, as shown by the relatively lower *I* ^2^ values for some of the high-risk cancer analyses, and more importantly, it allowed us to estimate the risk of VTE specifically in groups of patients most likely to benefit from anticoagulant intervention. The results presented in the review can only be generalised to the cancer types selected for investigation, which were selected on the basis of their high prevalence in Western countries or because of evidence of a strong association with VTE risk from previous research that used different study designs from those included here.

The main limitation of our work was that the decision to pool results was often hampered by high levels of heterogeneity occurring among the individual studies. In descriptive epidemiological studies, where a statistic is estimated among a single group (such as in this review), the potential for heterogeneity is far greater than for analytic or comparative studies (i.e., when two groups are compared to calculate a measure of effect such as an odds or risk ratio). This is because incidence rates are very sensitive to the choice of study population, plus other factors such as the definition of the outcome event and the duration of average follow-up, meaning at least some heterogeneity will be inevitable; other published meta-analyses of this type also report very high levels of heterogeneity [85],[86]. Despite this, five of our eight high-risk analyses were accompanied by *I* ^2^ values less than 85%, within the informally defined acceptable limit for carrying out meta-analyses under random effects.

The problem of heterogeneity was more evident when pooling results from the average-risk populations, where *I* ^2^ values exceeded 90% in eight of the nine analyses. This was due to a combination of very large cohort sizes (greatly minimising the level of within-study variance) and important differences with respect to study duration between the three cohorts that contributed the most data (Chew et al. [33], California; Blom et al. [30], Netherlands; Cronin-Fenton, et al. [36], Denmark). In the Dutch study, which considered events occurring in the 6 mo following diagnosis, VTE rates were always highest, followed by the Californian study, where the authors considered events over a 2-y period following diagnosis, with the lowest rates occurring in the Danish study, where average follow-up was usually longer than 2 y. This pattern would be expected, considering that VTE is most likely to occur immediately following cancer diagnosis because patients are more ill and more likely to receive high-risk treatments during the first 6 mo of their illness, as an earlier case-control study has shown [87]. This would account for the more comparable rates of VTE between the Dutch and Californian cohorts associated with cancers of the lung, brain, and pancreas, where, due to the poor prognosis of these cancer types, the median follow-up of patients was short and of similar length in both cohorts. Even allowing for the slightly longer duration of follow-up, rates of VTE were somewhat lower in the Danish national registry study [36], noticeably so for some cancers (i.e., brain cancer). It is probable that rates of VTE were underestimated in this report, partly as a result of the authors' only considering VTE events that were recorded in inpatient hospital records.

Differences in methods of VTE ascertainment could more generally be an important factor that explains many of the differences in VTE rates across this review as a whole. However, our restriction to symptomatic VTE will have alleviated this problem to some extent. Differences in the study populations with respect to geographical location and age could also be important sources heterogeneity. However, within the analysis of each cancer type, the average ages of the study participants were broadly similar. Overall, the small number of studies pooled in each analysis precluded us from carrying out a detailed statistical investigation to evaluate the relative importance of the different sources of heterogeneity.

Differences in use of anticoagulants by some or all of the participants in individual studies could further contribute to heterogeneity in the results. Where information on prophylaxis use in study participants was available, prophylaxis reflected existing guidelines, with anticoagulant prophylaxis given to medical inpatients [23],[53] and those undergoing surgical procedures for either brain [31],[58],[59], lung [28],[47], or prostate [55] tumours. In recent years, the trend towards shorter inpatient hospital stays and improved advice in terms of ambulation, in addition to prophylaxis, might have resulted in lower rates of VTE than in the past, especially among surgical patients. There were no older studies (prior to 1997) comprising cancer patients receiving surgery that met our review criteria, some of which were excluded because person-years of follow-up could not be calculated from the data presented in the paper. If older studies had been carried out that met our review criteria, then our pooled VTE rates (at least in the high-risk group) would inevitably be higher. Furthermore, two studies included in this review hypothesized that the reason surgical patients in their study had risks of VTE similar to those receiving alternative treatments or no treatment was because these patients received adequate levels of prophylaxis, thus compensating for their higher baseline risk [28],[60]. In contrast, the majority of studies where it was explicitly stated that patients did not receive prophylaxis were those comprising cancer patients receiving outpatient chemotherapy [37],[43],[44],[66]. In two additional studies of chemotherapy patients, whilst anticoagulant use among their sample was not specified, it was mentioned elsewhere in the paper that thromboprophylaxis was not the current standard of care for this group [19],[46]. Therefore, we believe our results reflect contemporary risks of VTE that exist among patients not receiving prophylaxis other than for short spells during hospital admission.

Our review of cohort studies has confirmed findings from previous studies using alternative study designs that cancers of the brain and pancreas are associated with the highest risk of VTE, but the increase in risk of VTE with haematological malignancy was less elevated, in contrast with earlier case-control data [87]. Of course, our focus on absolute risks would tend to downplay the influence of cancers that occur more commonly in younger patients, whose background risk of VTE is considerably lower. One additional finding of note was the very low risk of VTE in breast cancer among studies classed as average risk. This was in part due to inclusion of a study that reported a very low VTE rate of 0.2% a year in a large sample of women who had stage 1 or 2 breast cancer [40]. Finally, of the cancer types considered here, bone and soft malignancies were the least studied, with no studies in high-risk populations meeting our review criteria and only two in average-risk populations, which reported vastly different estimates of VTE risk [30],[36] (7 and 78 per 1,000 person-years). This highlights a dearth of research on the thromboembolic risk associated with this cancer type.

Our review highlights clearly that there are groups of patients who would be more suitable candidates to receive anticoagulant prophylaxis based on their underlying risk of VTE. Of course, our classification into “high” and “average” risk was done at the study rather than at the patient level. Only through primary studies could precise estimation of risks be stratified according to characteristics of the patient. Perhaps more important, though, will be the need to identify the within-person periods of greatest risk, including the time since diagnosis as discussed above, so that the timing of any anticoagulant intervention is most efficient.

Whilst identification of the periods of and persons at greatest risk is crucial, determining the threshold at which prophylaxis should be administered is beyond the scope of the present work, as other factors need consideration. One factor relates to the effectiveness of agents designed to prevent the occurrence of VTE. Anticoagulant agents, including both unfractionated and low-molecular-weight heparin and fondaparinux, are effective in the prevention of VTE, as shown in randomised controlled trials of acutely ill medical patients [88],[89]. Among patients exclusively with cancer, less clear evidence is available, with the exception of one trial that reported an 85% reduction in the risk of VTE among breast cancer patients with low-dose warfarin administration [90]. Another consideration is the bleeding risk known to exist with anticoagulants, particularly as these risks are believed to be higher in cancer patients [91],[92]. The need to balance the benefit against the risks of intervention would thus further affect the optimum threshold for intervening in this patient group.

In conclusion, by reviewing the existing literature we found that the risk of VTE is high in patients with cancer and varies markedly with cancer type. We believe that we have produced estimates of the risks of VTE in eight specific cancer types that are more accurate than those previously available. Future updates to clinical guidelines should incorporate estimates of absolute VTE risk obtained both from this paper and subsequent research, and should consider the targeting of VTE prophylaxis in the 6-mo period following cancer diagnosis, where the risk of a new VTE has been shown to be greatest.

## Supporting Information

Figure S1 **Pooled rates of venous thromboembolism per 1,000 person-years for studies where follow-up started at the time of cancer diagnosis.** Numbers in brackets refer to the number of studies that contributed to the pooled estimate.
(TIF)

Table S1 **Characteristics of included studies.**
(DOCX)

Table S2 **Risk of venous thromboembolism in cancer overall, with pooled incidence rates and 95% confidence intervals obtained from random effects meta-analysis.**
(DOCX)

Table S3 **Risk of venous thromboembolism in women with breast cancer, with pooled incidence rates and 95% confidence intervals obtained from random effects meta-analysis.**
(DOCX)

Table S4 **Risk of venous thromboembolism in people with lung cancer, with pooled incidence rates and 95% confidence intervals obtained from random effects meta-analysis.**
(DOCX)

Table S5 **Risk of venous thromboembolism in people with colorectal cancer, with pooled incidence rates and 95% confidence intervals obtained from random effects meta-analysis.**
(DOCX)

Table S6 **Risk of venous thromboembolism in men with prostate cancer, with pooled incidence rates and 95% confidence intervals obtained from random effects meta-analysis.**
(DOCX)

Table S7 **Risk of venous thromboembolism in people with brain cancer, with pooled incidence rates and 95% confidence intervals obtained from random effects meta-analysis.**
(DOCX)

Table S8 **Risk of venous thromboembolism in people with bone cancer, with pooled incidence rates and 95% confidence intervals obtained from random effects meta-analysis.**
(DOCX)

Table S9 **Risk of venous thromboembolism in people with haematological cancer, with pooled incidence rates and 95% confidence intervals obtained from random effects meta-analysis.**
(DOCX)

Table S10 **Risk of venous thromboembolism in people with pancreatic cancer, with pooled incidence rates and 95% confidence intervals obtained from random effects meta-analysis.**
(DOCX)

Text S1 **PRISMA checklist.**
(DOCX)

Text S2 **Medline search strategy.**
(DOCX)

Text S3 **Embase search strategy.**
(DOCX)

## Abbreviations

VTE: venous thromboembolism
