# Supplementary figures and images for: Risk of Venous Thromboembolism in Patients with Cancer: A Systematic Review and Meta-Analysis
Source: PLoS Med. 2012 Jul 31;9(7):e1001275. doi: 10.1371/journal.pmed.1001275 (PMC3409130; doi:10.1371/journal.pmed.1001275)

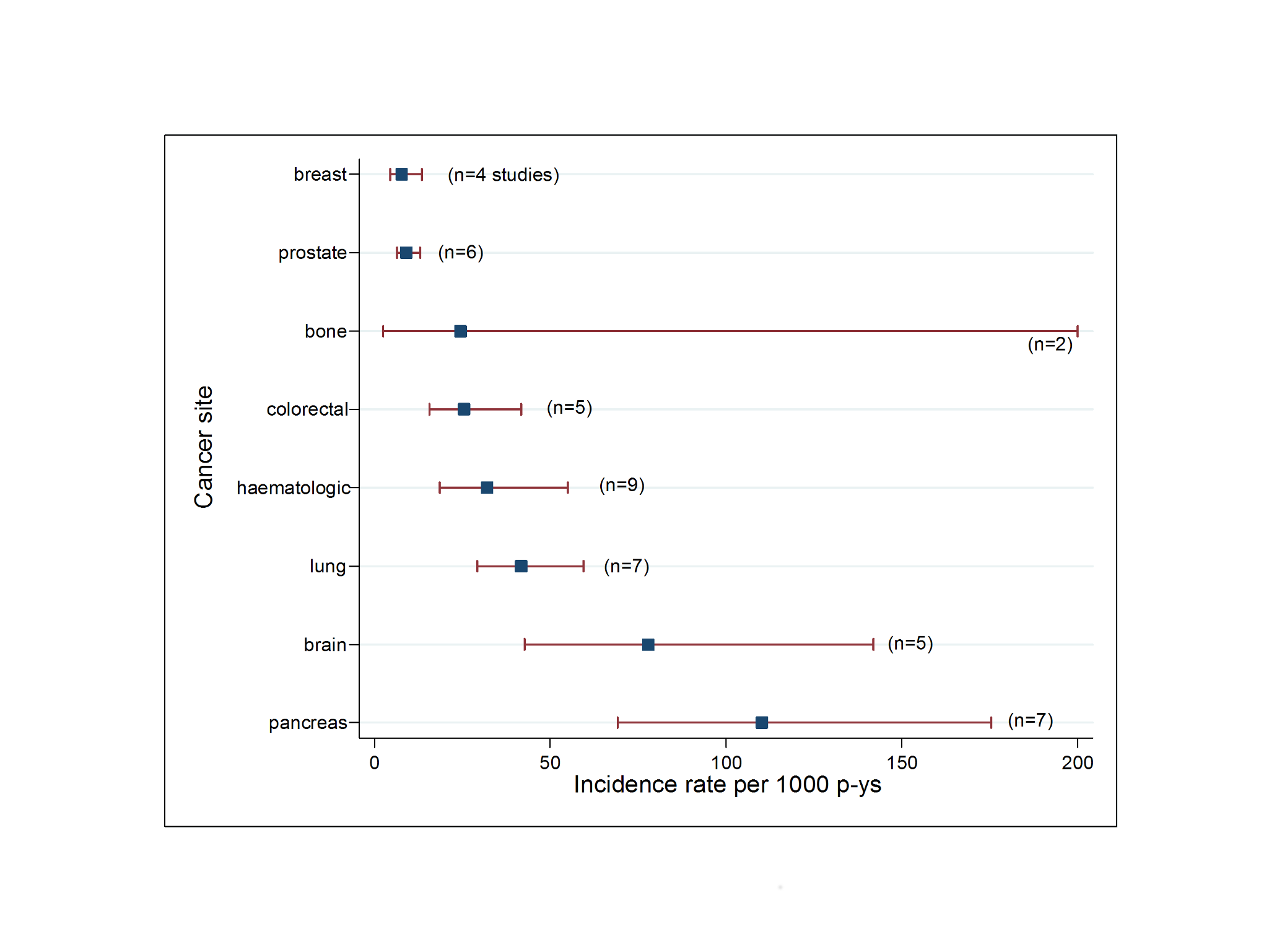

Supplement: Figure S1 — Pooled rates of venous thromboembolism per 1,000 person-years for studies where follow-up started at the time of cancer diagnosis. Numbers in brackets refer to the number of studies that contributed to the pooled estimate. (TIF) [file pmed.1001275.s001.tif]
