# Supplementary material for: Risk of Venous Thromboembolism in Patients with Cancer: A Systematic Review and Meta-Analysis
Source: PLoS Med. 2012 Jul 31;9(7):e1001275. doi: 10.1371/journal.pmed.1001275 (PMC3409130; doi:10.1371/journal.pmed.1001275)
Supplement: Table S1 — Characteristics of included studies. (DOCX) [file pmed.1001275.s002.docx]

Table S1: Characteristics of Included Studies

| First Author (Reference) | Country | Age (average ^a^)  Gender | Date(s) | Cohort size^b^ | Sample description | Cancer types | Outcome definition ^c^ | Risk |
| --- | --- | --- | --- | --- | --- | --- | --- | --- |
| Abdel-Razeq [23] | Jordan | 51 years 49% F | 2008 | 606 | Cancer patients admitted to medical units at the King Hussein Cancer Center | Overall | Any VTE | High  (hospitalised) |
| Andtbacka [[25](#_ENREF_25)] | U.S.A. | 54.4 years 99% F | 2000-2003 | 3,898 | Patients undergoing breast cancer surgery and treated according to clinical pathways | Breast | DVT or PE | High  (surgery) |
| Arai [[26](#_ENREF_26)] | Japan | 67 years 0% F | 1991-1998 | 638 | Patients who underwent radical retropubic prostatectomy at urological centres | Prostate | DVT or PE | High  (surgery) |
| Auguste [[27](#_ENREF_27)] | U.S.A | 47 years 42% F | 1997-2000 | 180 | All patients undergoing craniotomy with motor mapping for glioma | Brain | DVT or PE | High  (surgery) |
| Ay [[20](#_ENREF_20)]  (Includes data extracted from Kanz [41] and Vormittag [62]) | Austria | 62 years 45% F | 2003-2008 | 821 | Patients with newly diagnosed cancer or disease progression after complete or partial remission | Overall, breast, lung, colorectal, prostate, brain, pancreas, haematological | Any VTE | High  (various treatments) |
| Blom [[30](#_ENREF_30)] | Holland | 67 years 50% F | 1986-2002 | 66,329 | Population-based cancer registry data linked to hospital admission records and anticoagulant clinic data | Overall, breast, lung, colorectal, prostate, brain, bone, pancreas, haematological | Any VTE | Average |
| Blom [[28](#_ENREF_28)] | Holland | 65 years 20% F | 1990-2000 | 537 | Consecutive patients admitted with non-small cell lung cancer | Lung | DVT or PE | High  (various treatments) |
| Blom [[29](#_ENREF_29)] | Holland | 64 years 43% F | 1990-2000 | 202 | Consecutive patients with tumour of the pancreas | Pancreas | DVT or PE | High  (high grade disease) |
| Brandes [[31](#_ENREF_31)] | Italy | 52 years 53% F | Not stated | 77 | Patients aged 18-70 years undergoing surgery for high grade glioma | Brain | DVT or PE | High  (surgery) |
| Chew [[33](#_ENREF_33)]  (Includes data extracted from Chew [[34](#_ENREF_34)], Chew [[32](#_ENREF_32)], Alcalay [[24](#_ENREF_24)], Semrad [56] and Ku [45]) | U.S.A | 65 years 49% F | 1993-1995 | 235,149 | California Cancer Registry linked to the California Patient Discharge Data Set | Overall, breast, lung, colorectal prostate, brain, pancreatic, haematological. | DVT or PE | Average |
| Cronin-Fenton [[36](#_ENREF_36)] | Denmark | all aged >15 yrs; 48% F | 1997-2005 | 57,591 | Individually linked data between Danish National Registry of Patients, Danish Civil Registration System and Data Civil Registration System | Overall, breast, lung, colorectal, prostate, brain, bone, pancreas, haematological | Any VTE | Average |
| Di Nisio [38] | Italy | 59 yrs 62% F | 2003-2009 | 1,921 | Cancer patients starting chemotherapy at a single institution | Overall | DVT or PE | High  (chemotherapy) |
| De Stefano [[37](#_ENREF_37)] | Italy | 60 years 53% F | 1994-2003 | 379 | Patients with acute leukaemia admitted to a single institution | Haematological | Any VTE | High  (various treatments) |
| Hall [39] | U.S.A | 75 years 47% F | 1995-1999 | 14,214 | Patients with stage III (or metastatic) cancers of the breast, lung, prostate, colon or pancreas selected from the SEER-Medicare database | Overall, breast, colorectal, lung, prostate, pancreas | DVT or PE | High  (high grade disease) |
| Hernandez [40] | Denmark | age range: 45-69 years 100% F | 1990-2004 | 16,289 | Women with stage I or II breast cancer selected from the Danish Breast Cancer Cooperative Group (DBCG) clinical database. | Breast | DVT or PE | Average |
| Kauffman [42] | U.S.A. | 58 years 48% F | 2005-2007 | 44 | Patients with symptomatic multiple myeloma receiving triple therapy (bortezomib, thalidomide and dexamaethasone) at a single centre in the US | Haematological | DVT only | High  (various treatments) |
| Khorona [[19](#_ENREF_19)]  (Includes data from Connolly [[35](#_ENREF_35)]) | U.S.A | 60 years 67% F | 2002-2004 | 4,405 | Patients initiating a new chemotherapy regime enrolled in the Awareness of Neutropenia in Cancer study. | Overall, breast, lung, colorectal, haematological | DVT or PE | High  (chemotherapy) |
| Kirwan [43] | U.K. | 52 years 100% F | not stated | 123 | Women commencing chemotherapy following a diagnosis of breast cancer. | Breast | VTE (not stated) | High  (chemotherapy) |
| Komrokji [44] | U.S.A | 55 years 44% F | 1990-2001 | 201 | Patients with a new diagnosis of diffuse large B-cell lymphoma receiving chemotherapy | Haematological | Any VTE | High  (chemotherapy) |
| Mandala [46] | Italy | Range: 26-85 years 69% F | 2003-2006 | 381 | Consecutive patients receiving adjuvant chemotherapy. | Breast, colorectal | Any VTE | High  (chemotherapy) |
| Mason [47] | U.S.A. | 61 years; 26% F | 1990-2001 | 336 | Patients undergoing pneumectomy for lung cancer at a single clinic in Ohio | lung | DVT or PE | High  (surgery) |
| Negaard [48] | Norway | 62 years 43% F | 2004-2006 | 93 | Patient with various haematological malignancies recruited from a University Hospital department | Haematological | DVT or PE | High  (various treatments) |
| Numico [49] | Italy | 64 years 21% F | 2000-2003 | 108 | Patients with nonsmall cell lung carcinoma (NSCLC) consecutively recruited from a single centre | Lung | DVT or PE | High  (chemotherapy) |
| Oh [50] | Korea | 67 years 41% F | 2003-2005 | 75 | Newly diagnosed patients admitted to hospital with advanced pancreatic adenocarcinoma | Pancreas | Any VTE | High  (high grade disease) |
| Otten [51] | Holland | 58 years 56% F | 1995-2000 | 206 | Consecutive patients  with malignancy treated with chemotherapy | Overall | Any VTE | High  (chemotherapy) |
| Poruk [52] | U.S.A | 68 years 45% F | not stated | 133 | Patients with histologically or cytologically confirmed pancreatic ductal adenocarcinoma | Pancreas | Any VTE | High  (high grade disease) |
| Reeves [53] | U.S.A | 58 years; 55% F | 2007 | 176 | Cancer patients admitted to a single inpatient institution (Karmonas cancer center) | Overall | DVT or PE | High  (hospitalised) |
| Sallah [54] | U.S.A | 60 years 46% F | 1993-2000 | 1,041 | Patients referred to haematology/oncology services for cancer treatment | Overall | DVT or PE | High  (various treatments) |
| Secin [55] | Europe and U.S.A. | 62 years 0% F | 1995-2006 | 5,951 | Prostate cancer patients undergoing laparascopic surgery | Prostate | DVT or PE | High  (surgery) |
| Sgarabotto [57] | Italy | 60 years 50% F | 1986-1996 | 415 | Consecutive patients referred to an outpatients’ clinic | Haematological | Any VTE | Average |
| Simanek [58] | Austria | 58 years 57% F | 2003-2004 | 63 | Consecutive patients with high grade glioma at a single oncology department. | Brain | DVT or PE | High  (surgery) |
| Streiff [59] | U.S.A. | 55 years 36% F | 1991-2001 | 130 | Adult patients undergoing operation for malignant glioma | Brain | DVT or PE | High  (surgery) |
| Tagalakis [60] | Canada | 65 years 40% F | 1997-2004 | 493 | Patients with histologically confirmed non-small cell lung cancer | Lung | DVT only | High  (high grade disease) |
| Van Hemelrijck [61] | Sweden | 62% aged >65 years 0% F | 1997-2007 | 76,600 | Newly diagnosed cases of prostate cancer, based on data from the National Prostate Cancer Registry | Prostate | DVT or PE | Average |
| Weder [63] | Switzerland | 56 years 31% F | 1998 - 2007 | 176 | Patients with locally advanced non-small cell lung cancer who were treated with neoadjuvant, chemotherapy and radiotherapy followed by pneumonectomy | Lung | PE only | High  (various treatments) |
| Whittle [64] | U.K. | 64 years 38% F | 2005- | 268 | Patients referred to or followed-up at clinic for chronic lymphocytic leukaemia in Hull, East Yorkshire | Haematological | DVT or PE | Average |
| Zechinna [65] | Italy | 66 years 16% F | not stated | 49 | Patients receiving systemic chemotherapy for locally advanced or metastatic lung cancer | Lung | Any VTE | High  (chemotherapy) |
| Zhou [66] | U.S.A | 57 years 22% F | 2003 | 422 | Patients referred to lymphoma department of Anderson Cancer Center and receiving at least 1 cycle of chemotherapy | Haematological | DVT or PE | High  (chemotherapy) |

DVT deep vein thrombosis; PE pulmonary embolism
a Mean or median as specified in paper
b Cohort size used in the analysis of overall cancer for studies where results were extracted from multiple reports.
c Outcome was defined as DVT or PE if all outcome events reported in the paper (when specified) were either deep vein thrombosis of the lower or upper extremities or pulmonary embolism even if other types of VTE would have been included had they occurred. Outcome was defined as Any VTE when other types of VTE including superficial vein thrombosis, portal vein thrombosis, retinal vein thrombosis, sinus vein thrombosis, inferior vena cava thrombosis and mesenteric vein thrombosis were included.
