# Supplementary material for: Risk of Venous Thromboembolism in Patients with Cancer: A Systematic Review and Meta-Analysis
Source: PLoS Med. 2012 Jul 31;9(7):e1001275. doi: 10.1371/journal.pmed.1001275 (PMC3409130; doi:10.1371/journal.pmed.1001275)
Supplement: Table S2 — Risk of venous thromboembolism in cancer overall, with pooled incidence rates and 95% confidence intervals obtained from random effects meta-analysis. (DOCX) [file pmed.1001275.s003.docx]

Table S2: Risk of venous thromboembolism in cancer overall^a^ with pooled incidence rates and 95% confidence intervals obtained from random effects meta-analysis.

| First author (year)[ref] | No. of participants | Total person-years of follow-up | No. of people with VTE | Incidence rate/1000 person-years (95% confidence interval)^b^ | Average follow-up duration^c^ (months) |
| --- | --- | --- | --- | --- | --- |
| **Average risk** |  |  |  |  |  |
| Blom (2006)[[30](#_ENREF_30)] | 66,329 | 31,867 | 815 | 25.6 (23.9, 27.4) | 6 |
| Chew (2006)[[33](#_ENREF_33)] | 235,149 | 389,150 | 3,775 | 9.7 (9.4, 10.0) | 20 |
| Cronin-Fenton (2010)[[36](#_ENREF_36)] | 57,591 | 127,492 | 1,023 | 8.0 (7.6, 9.5) | 27 |
| Pooled incidence rate |  |  |  | **12.6 (7.0, 22.6)** |  |
| Heterogeneity (I ² =99.7%) |  |  |  |  |  |
| **High risk** |  |  |  |  |  |
| Sallah (2002)[54] | 1,041 | 2,256 | 81 | 35.9 (28.9, 44.7) | 26 |
| Otten (2004)[51] | 206 | 133.1 | 15 | 112.7 (67.9, 186.9) | 8 |
| Ay (2009)[[20](#_ENREF_20)] | 821 | 1,126 | 62 | 55.1 (42.9, 70.6) | 16 |
| Hall (2009)[39] | 14,214 | 9,249 | 489 | 52.9 (48.4, 57.8) | 8 |
| Connolly (2010)[[35](#_ENREF_35)] | 4,405 | 904.5 | 93 | 102.8 (83.9, 126.0) | 2 |
| Abdel-Razaq (2010)[[23](#_ENREF_23)] | 606 | 111.0 | 21 | 189.2 (123.4, 290.2) | 2 |
| Di Nisio (2010)[38] | 1,921 | 1,281 | 39 | 30.5 (22.3, 41.7) | 8 |
| Reeves (2010)[53] | 176 | 17.2 | 2 | 116.3 (29.1, 464.9) | 1 |
| Pooled incidence rate |  |  |  | **68.0 (48.0, 96.4)** |  |
| Heterogeneity (I ² =93.4%) |  |  |  |  |  |

a Combined data from cancer types which in total account for 75% or more of all cancer diagnoses based on UK figures.
b Studies pooled using random effects meta-analysis.
c Mean duration of follow-up, except where this was not stated or could not be calculated in which case the median was used.
