# Supplementary material for: Risk of Venous Thromboembolism in Patients with Cancer: A Systematic Review and Meta-Analysis
Source: PLoS Med. 2012 Jul 31;9(7):e1001275. doi: 10.1371/journal.pmed.1001275 (PMC3409130; doi:10.1371/journal.pmed.1001275)
Supplement: Table S3 — Risk of venous thromboembolism in women with breast cancer, with pooled incidence rates and 95% confidence intervals obtained from random effects meta-analysis. (DOCX) [file pmed.1001275.s004.docx]

Table S3: Risk of venous thromboembolism in women with breast cancer with pooled incidence rates and 95% confidence intervals obtained from random effects meta-analysis.

| First author (year)[ref] | No. of participants | Total person-years of follow-up | No. of people with VTE | Incidence rate/1000 person-years (95% confidence interval)^a^ | Average follow-up duration^b^ (months) |
| --- | --- | --- | --- | --- | --- |
| **Average risk** |  |  |  |  |  |
| Blom (2006)[[30](#_ENREF_30)] | 10,556 | 5,199 | 84 | 16.2 (13.1, 20.0) | 6 |
| Chew (2007)[[34](#_ENREF_34)] | 108,255 | 205,270 | 1,299 | 6.3 (6.0, 6.7) | 23 |
| Hernandez (2009)[40] | 16,289 | 65,156 | 137 | 2.1 (1.8, 2.5) | 48 |
| Cronin-Fenton (2010))[[36](#_ENREF_36)] | 8,586 | 30,391 | 119 | 3.9 (3.3, 4.7) | 42 |
| Pooled incidence rate |  |  |  | **5.4 (2.8, 10.3)** |  |
| Heterogeneity (I ² =98.8%) |  |  |  |  |  |
| **High risk** |  |  |  |  |  |
| Khorana (2005)[[19](#_ENREF_19)] | 1,074 | 214.8 | 16 | 74.5 (45.6, 121.6) | 2 |
| Andtbacka (2006)[[25](#_ENREF_25)] | 3,898 | 639.4 | 7 | 10.9 (5.2, 23.0) | 2 |
| Kirwan (2008)[43] | 123 | 30.8 | 8 | 259.7 (129.9, 519.4) | 3 |
| Hall (2009)[39] | 1,789 | 1,549 | 72 | 46.5 (36.9, 58.6) | 10 |
| Mandala (2010)[46] | 182 | 91 | 16 | 175.8 (107.7, 287.0) | 6 |
| Kanz (2011)[41] | 144 | 273.7 | 3 | 11.0 (3.5, 34.0) | 23 |
| Pooled incidence rate |  |  |  | **55.1 (24.9, 122.0)** |  |
| Heterogeneity (I ² =92.9%) |  |  |  |  |  |

a Studies pooled using random effects meta-analysis.
b Mean duration of follow-up, except where this was not stated or could not be calculated in which case the median was used.
