# Supplementary material for: Risk of Venous Thromboembolism in Patients with Cancer: A Systematic Review and Meta-Analysis
Source: PLoS Med. 2012 Jul 31;9(7):e1001275. doi: 10.1371/journal.pmed.1001275 (PMC3409130; doi:10.1371/journal.pmed.1001275)
Supplement: Table S4 — Risk of venous thromboembolism in people with lung cancer, with pooled incidence rates and 95% confidence intervals obtained from random effects meta-analysis. (DOCX) [file pmed.1001275.s005.docx]

Table S4: Risk of venous thromboembolism in people with lung cancer with pooled incidence rates and 95% confidence intervals obtained from random effects meta-analysis.

| First author (year)[ref] | No. of participants | Total person-years of follow-up | No. of people with VTE | incidence rate/1000 person-years (95% confidence interval)^a^ | Average follow-up duration^b^ (months) |
| --- | --- | --- | --- | --- | --- |
| **Average risk** |  |  |  |  |  |
| Blom (2006)[[30](#_ENREF_30)] | 9,336 | 3,651 | 129 | 35.3 (29.7, 42.0) | 5 |
| Chew (2008)[[32](#_ENREF_32)] | 91,933 | 93,073 | 3,140 | 33.7 (32.6, 34.9) | 12 |
| Cronin-Fenton (2010)[[36](#_ENREF_36)] | 7,975 | 7,872 | 127 | 16.1 (13.6, 19.2) | 12 |
| Pooled incidence rate |  |  |  | **26.9 (17.6, 41.1)** |  |
| Heterogeneity (I ² =97.0%) |  |  |  |  |  |
| **High risk** |  |  |  |  |  |
| Blom (2004)[[28](#_ENREF_28)] | 537 | 879 | 39 | 44.4 (32.2, 60.7) | 20 |
| Khorana (2005)[[19](#_ENREF_19)] | 574 | 114.8 | 16 | 139.4 (85.4, 227.5) | 2 |
| Numico (2005)[49] | 108 | 78.3 | 12 | 153.3 (87.0, 269.9) | 9 |
| Mason (2006)[47] | 336 | 828 | 25 | 30.2 (20.4, 44.7) | 30 |
| Tagalakis (2007)[60] | 493 | 634 | 67 | 105.7 (83.2, 134.3) | 15 |
| Zecchina (2007)[65] | 49 | 53 | 3 | 56.6 (18.3, 175.5) | 13 |
| Hall (2009)[39] | 8,102 | 4,449 | 273 | 61.4 (54.5, 69.1) | 7 |
| Weder (2010)[63] | 176 | 41.9 | 3 | 71.6 (23.1, 222.0) | 3 |
| Kanz (2011)[41] | 142 | 141.2 | 6 | 42.5 (19.1, 94.6) | 12 |
| Pooled incidence rate |  |  |  | **68.7 (49.1, 96.3)** |  |
| Heterogeneity (I ² =85.8%) |  |  |  |  |  |

a Studies pooled using random effects meta-analysis.
b Mean duration of follow-up, except where this was not stated or could not be calculated in which case the median was used.
