# Supplementary material for: Risk of Venous Thromboembolism in Patients with Cancer: A Systematic Review and Meta-Analysis
Source: PLoS Med. 2012 Jul 31;9(7):e1001275. doi: 10.1371/journal.pmed.1001275 (PMC3409130; doi:10.1371/journal.pmed.1001275)
Supplement: Table S5 — Risk of venous thromboembolism in people with colorectal cancer, with pooled incidence rates and 95% confidence intervals obtained from random effects meta-analysis. (DOCX) [file pmed.1001275.s006.docx]

Table S5: Risk of venous thromboembolism in people with colorectal cancer with pooled incidence rates and 95% confidence intervals obtained from random effects meta-analysis.

| First author (year)[ref] | No. of participants | Total person-years of follow-up | No. of people with VTE | incidence rate/1000 person-years (95% confidence interval)^a^ | Average follow-up duration^b^ (months) |
| --- | --- | --- | --- | --- | --- |
| **Average risk** |  |  |  |  |  |
| Blom (2006)[[30](#_ENREF_30)] | 8,286 | 3,932 | 102 | 25.9 (21.4, 31.5) | 6 |
| Alcalay (2006)[[24](#_ENREF_24)] | 68,042 | 110,331 | 2,100 | 19.0 (18.2, 19.9) | 19 |
| Cronin-Fenton (2010)[36] | 8,373 | 20,613 | 181 | 8.8 (7.6, 10.2) | 30 |
| Pooled incidence rate |  |  |  | **16.3 (9.6, 27.7)** |  |
| Heterogeneity (I ² =98.2%) |  |  |  |  |  |
| **High risk** |  |  |  |  |  |
| Khorana (2005))[[19](#_ENREF_19)] | 323 | 64.6 | 3 | 46.4 (15.0, 144.0) | 2 |
| Hall (2009)[39] | 1,890 | 1,568 | 74 | 47.2 (37.6, 59.3) | 10 |
| Mandala (2010)[46] | 129 | 69.0 | 9 | 130.4 (67.9, 250.7) | 6 |
| Kanz (2011)[41] | 116 | 163.8 | 11 | 67.2 (37.2, 121.3) | 17 |
| Pooled incidence rate |  |  |  | **65.7 (40.1, 107.5)** |  |
| Heterogeneity (I ² =66.4%) |  |  |  |  |  |

a Studies pooled using random effects meta-analysis.
b Mean duration of follow-up, except where this was not stated or could not be calculated in which case the median was used.
