# Supplementary material for: Risk of Venous Thromboembolism in Patients with Cancer: A Systematic Review and Meta-Analysis
Source: PLoS Med. 2012 Jul 31;9(7):e1001275. doi: 10.1371/journal.pmed.1001275 (PMC3409130; doi:10.1371/journal.pmed.1001275)
Supplement: Table S6 — Risk of venous thromboembolism in men with prostate cancer, with pooled incidence rates and 95% confidence intervals obtained from random effects meta-analysis. (DOCX) [file pmed.1001275.s007.docx]

Table S6: Risk of venous thromboembolism in men with prostate cancer with pooled incidence rates and 95% confidence intervals obtained from random effects meta-analysis.

| First author (year)[ref] | No. of participants | Total person-years of follow-up | No. of people with VTE | incidence rate/1000 person-years (95% confidence interval)^a^ | Average follow-up duration^b^ (months) |
| --- | --- | --- | --- | --- | --- |
| **Average risk** |  |  |  |  |  |
| Blom (2006)[[30](#_ENREF_30)] | 6,013 | 2,862 | 57 | 19.9 (15.4, 25.8) | 6 |
| Chew (2006)[[33](#_ENREF_33)] | 43,939 | 83,816 | 460 | 5.5 (5.0, 6.0) | 23 |
| Cronin-Fenton (2010)[[36](#_ENREF_36)] | 4,457 | 9,757 | 98 | 10.0 (8.2, 12.2) | 26 |
| Van Hemelrijck (2010)[61] | 76,600 | 315,320 | 1,640 | 5.2 (5.0, 5.5) | 49 |
| Pooled incidence rate |  |  |  | **8.5 (5.8, 12.3)** |  |
| Heterogeneity (I ² =97.8%) |  |  |  |  |  |
| **High risk** |  |  |  |  |  |
| Arai (2000)[[26](#_ENREF_26)] | 638 | 52.4 | 3 | 57.3 (18.5, 177.5) | 1 |
| Secin (2008)[55] | 5,951 | 1,466 | 31 | 21.1 (14,9, 30.1) | 3 |
| Hall (2009)[39] | 1,596 | 1,378 | 20 | 14.5 (9.4, 22.5) | 10 |
| Kanz (2011)[41] | 113 | 208.2 | 1 | 4.8 (0.7, 34.1) | 22 |
| Pooled incidence rate |  |  |  | **19.5 (11.5, 33.0)** |  |
| Heterogeneity (I ² =59.2%) |  |  |  |  |  |

a Studies pooled using random effects meta-analysis.
b Mean duration of follow-up, except where this was not stated or could not be calculated in which case the median was used.
