# Supplementary material for: Risk of Venous Thromboembolism in Patients with Cancer: A Systematic Review and Meta-Analysis
Source: PLoS Med. 2012 Jul 31;9(7):e1001275. doi: 10.1371/journal.pmed.1001275 (PMC3409130; doi:10.1371/journal.pmed.1001275)
Supplement: Table S7 — Risk of venous thromboembolism in people with brain cancer, with pooled incidence rates and 95% confidence intervals obtained from random effects meta-analysis. (DOCX) [file pmed.1001275.s008.docx]

Table S7: Risk of venous thromboembolism in people with brain cancer with pooled incidence rates and 95% confidence intervals obtained from random effects meta-analysis.

| First author (year)[ref] | No. of participants | Total person-years of follow-up | No. of people with VTE | incidence rate/1000 person-years (95% confidence interval)^a^ | Average follow-up duration^b^ (months) |
| --- | --- | --- | --- | --- | --- |
| **Average risk** |  |  |  |  |  |
| Blom (2006)[[30](#_ENREF_30)] | 1,058 | 381.6 | 34 | 89.1 (63.7, 124.7) | 4 |
| Semrad (2007)[56] | 9,489 | 11,107 | 715 | 64.4 (59.8, 69.3) | 14 |
| Cronin-Fenton (2010)[[36](#_ENREF_36)] | 1,133 | 1,071 | 19 | 17.7 (11.3, 27.8) | 11 |
| Pooled incidence rate |  |  |  | **48.0 (24.4, 94.2)** |  |
| Heterogeneity (I ² =94.3%) |  |  |  |  |  |
| **High risk** |  |  |  |  |  |
| Brandes (1997)[[31](#_ENREF_31)] | 77 | 110.2 | 20 | 181.5 (117.1, 281.3) | 17 |
| Auguste (2003)[[27](#_ENREF_27)] | 180 | 20.8 | 6 | 288.5 (129.6, 642.1) | 1 |
| Streiff (2004) [59] | 130 | 130.0 | 28 | 215.4 (148.7, 311.9) | 12 |
| Simanek (2007)[58] | 63 | 60.0 | 15 | 249.9 (150.7, 414.6) | 11 |
| Vormittag (2009)[62] | 103 | 122.9 | 18 | 146.4 (92.2, 232.4) | 14 |
| Pooled incidence rate |  |  |  | **200.1 (162.2, 246.9)** |  |
| Heterogeneity (I ² =0.0%) |  |  |  |  |  |

a Studies pooled using random effects meta-analysis.
b Mean duration of follow-up, except where this was not stated or could not be calculated in which case the median was used.
