# Supplementary material for: Risk of Venous Thromboembolism in Patients with Cancer: A Systematic Review and Meta-Analysis
Source: PLoS Med. 2012 Jul 31;9(7):e1001275. doi: 10.1371/journal.pmed.1001275 (PMC3409130; doi:10.1371/journal.pmed.1001275)
Supplement: Table S8 — Risk of venous thromboembolism in people with bone cancer, with pooled incidence rates and 95% confidence intervals obtained from random effects meta-analysis. (DOCX) [file pmed.1001275.s009.docx]

Table S8: Risk of venous thromboembolism in people with bone cancer with pooled incidence rates and 95% confidence intervals obtained from random effects meta-analysis.

| First author (year)[ref] | No. of participants | Total person-years of follow-up | No. of people with VTE | incidence rate/1000 person-years (95% confidence interval)^a^ | Average follow-up duration^b^ (months) |
| --- | --- | --- | --- | --- | --- |
| **Average risk** |  |  |  |  |  |
| Blom (2006)[[30](#_ENREF_30)] | 159 | 77.1 | 6 | 77.8 (35.0, 173.3) | 6 |
| Cronin-Fenton (2010)[[36](#_ENREF_36)] | 229 | 541 | 4 | 7.4 (2.8, 19.7) | 28 |
| Pooled incidence rate |  |  |  | **24.4 (2.4, 245.2)** |  |
| Heterogeneity (I ² =92.5%) |  |  |  |  |  |

a Studies pooled using random effects meta-analysis.
b Mean duration of follow-up, except where this was not stated or could not be calculated in which case the median was used.
There were no studies classed as high risk which provided data on bone cancer.
