# Supplementary material for: Risk of Venous Thromboembolism in Patients with Cancer: A Systematic Review and Meta-Analysis
Source: PLoS Med. 2012 Jul 31;9(7):e1001275. doi: 10.1371/journal.pmed.1001275 (PMC3409130; doi:10.1371/journal.pmed.1001275)
Supplement: Table S9 — Risk of venous thromboembolism in people with haematological cancer, with pooled incidence rates and 95% confidence intervals obtained from random effects meta-analysis. (DOCX) [file pmed.1001275.s010.docx]

Table S9: Risk of venous thromboembolism in people with haematological cancer with pooled incidence rates and 95% confidence intervals obtained from random effects meta-analysis.

| First author (year)[ref] | No. of participants | Total person-years of follow-up | No. of people with VTE | incidence rate/1000 person-years (95% confidence interval)^a^ | Average follow-up duration^b^ (months) |
| --- | --- | --- | --- | --- | --- |
| **Average risk** |  |  |  |  |  |
| Sgarabotto (1998)[57] | 415 | 1,141 | 16 | 14.0 (8.6, 22.9) | 33 |
| Blom (2006)[[30](#_ENREF_30)] | 5,381 | 2,458 | 88 | 35.8 (29.0, 44.1) | 5 |
| Chew (2006)[[33](#_ENREF_33)] | 8,699 | 13,213 | 190 | 14.4 (12.5, 16.6) | 18 |
| Ku (2009)[45] | 7,876 | 7,494 | 394 | 52.6 (47.6, 58.0) | 11 |
| Cronin-Fenton (2010)[[36](#_ENREF_36)] | 4,498 | 10,023 | 120 | 12.0 (10.0, 14.3) | 27 |
| Whittle (2011)[64] | 268 | 966 | 14 | 14.5 (8.6, 24.5) | 43 |
| Pooled incidence rate |  |  |  | **20.3 (10.8, 38.4)** |  |
| Heterogeneity (I ² =98.6%) |  |  |  |  |  |
| **High risk** |  |  |  |  |  |
| Khorana (2005)[[19](#_ENREF_19)] | 316 | 63.2 | 8 | 126.6 (63.3, 253.1) | 2 |
| De Stefano (2005)[[37](#_ENREF_37)] | 379 | 439.3 | 11 | 25.0 (13.9, 45.2) | 14 |
| Komrokji (2006)[44] | 201 | 100.5 | 17 | 169.2 (105.2, 272.1) | 6 |
| Negaard (2008)[48] | 93 | 186 | 8 | 43.0 (21.5, 86.0) | 24 |
| Ay (2009)[[20](#_ENREF_20)] | 111 | 152.3 | 7 | 46.0 (21.9, 96.4) | 16 |
| Zhou (2010)[66] | 422 | 844 | 72 | 85.3 (67.7, 107.5) | 24 |
| Kauffman (2010)[42] | 44 | 91.7 | 2 | 21.8 (5.5, 87.2) | 25 |
| Pooled incidence rate |  |  |  | **62.6 (37.3, 105.0)** |  |
| Heterogeneity (I ² =82.8%) |  |  |  |  |  |

a Studies pooled using random effects meta-analysis.
b Mean duration of follow-up, except where this was not stated or could not be calculated in which case the median was used.
The studies reported by Chew [[33](#_ENREF_33)](non-Hodgkin lymphoma) and Ku [45](leukaemia).were both from the California Cancer Registry cohort. However, because they included different subsets of patients and were conducted over different time intervals (Chew, 1993-5 and Ku, 1993-9) a decision was made to pool these as separate studies
