# Supplementary material for: Risk of Venous Thromboembolism in Patients with Cancer: A Systematic Review and Meta-Analysis
Source: PLoS Med. 2012 Jul 31;9(7):e1001275. doi: 10.1371/journal.pmed.1001275 (PMC3409130; doi:10.1371/journal.pmed.1001275)
Supplement: Table S10 — Risk of venous thromboembolism in people with pancreatic cancer, with pooled incidence rates and 95% confidence intervals obtained from random effects meta-analysis. (DOCX) [file pmed.1001275.s011.docx]

Table S10: Risk of venous thromboembolism in people with pancreatic cancer with pooled incidence rates and 95% confidence intervals obtained from random effects meta-analysis.

| First author (year)[ref] | No. of participants | Total person-years of follow-up | No. of people with VTE | incidence rate/1000 person-years (95% confidence interval)^a^ | Average follow-up duration^b^ (months) |
| --- | --- | --- | --- | --- | --- |
| **Average risk** |  |  |  |  |  |
| Blom (2006)[[30](#_ENREF_30)] | 1,674 | 520.4 | 38 | 73.0 (53.1, 100.4) | 4 |
| Chew (2006)[33] | 5,289 | 3,550 | 231 | 65.1 (57.2, 74.0) | 8 |
| Cronin-Fenton (2010)[[36](#_ENREF_36)] | 1,671 | 881 | 36 | 40.9 (29.5, 56.7) | 6 |
| Pooled incidence rate |  |  |  | **58.9 (44.1, 78.6)** |  |
| Heterogeneity (I ² =74.4%) |  |  |  |  |  |
| **High risk** |  |  |  |  |  |
| Blom (2006a)[[29](#_ENREF_29)] | 202 | 176 | 19 | 108.0 (68.9, 169.3) | 10 |
| Oh (2008)[50] | 75 | 25.5 | 4 | 156.9 (58.9, 418.0) | 4 |
| Hall (2009)[39] | 837 | 305 | 50 | 164.0 (124.3, 216.4) | 4 |
| Poruk (2010)[52] | 133 | 118 | 20 | 169.5 (109.4, 262.7) | 11 |
| Kanz (2011)[41] | 63 | 57.8 | 11 | 190.5 (105.5, 343.9) | 11 |
| Pooled incidence rate |  |  |  | **155.1 (128.0, 187.9)** |  |
| Heterogeneity (I ² =0.0%) |  |  |  |  |  |

a Studies pooled using random effects meta-analysis.
b Mean duration of follow-up, except where this was not stated or could not be calculated in which case the median was used.
