# Supplementary material for: Risk of Venous Thromboembolism in Patients with Cancer: A Systematic Review and Meta-Analysis
Source: PLoS Med. 2012 Jul 31;9(7):e1001275. doi: 10.1371/journal.pmed.1001275 (PMC3409130; doi:10.1371/journal.pmed.1001275)
Supplement: Text S2 — Medline search strategy. (DOCX) [file pmed.1001275.s013.docx]

**Text S2: Medline search strategy**

1. Epidemiologic studies/
2. exp Case-Control Studies/
3. exp Cohort Studies/
4. case control.tw.
5. (cohort adj (study or studies)).tw.
6. cohort analy$.tw.
7. (Follow up adj (study or studies)).tw.
8. (observational adj (study or studies)).tw.
9. longitudinal.tw.
10. retrospective.tw.
11. Incidence/
12. 1 or 2 or 3 or 4 or 5 or 6 or 7 or 8 or 9 or 10 or 11
13. exp Venous Thrombosis/
14. exp Venous Thromboembolism/
15. exp Thrombosis/
16. exp Pulmonary Embolism/
17. (dvt* or (deep$ adj8 (vein$ or ven$) adj8 thromb$) or embol$).mp. [mp=title, original title, abstract, name of substance word, subject heading word, unique identifier]
18. 13 or 14 or 15 or 16 or 17
19. Carcinoma/
20. malig$.tw.
21. neoplas$.tw.
22. oncol$.tw.
23. tumo?r$.tw.
24. Neoplasms/
25. exp Brain Neoplasms/
26. exp Bone Neoplasms/
27. exp Pancreatic Neoplasms/
28. exp Lung Neoplasms/
29. exp Colorectal Neoplasms/
30. exp Breast Neoplasms/
31. exp Prostatic Neoplasms/
32. exp Hematologic Neoplasms/
33. 19 or 20 or 21 or 22 or 23 or 24 or 25 or 26 or 27 or 28 or 29 or 30 or 31 or 32
34. 12 and 18 and 33

limit 34 to humans
