# Supplementary material for: Risk of Venous Thromboembolism in Patients with Cancer: A Systematic Review and Meta-Analysis
Source: PLoS Med. 2012 Jul 31;9(7):e1001275. doi: 10.1371/journal.pmed.1001275 (PMC3409130; doi:10.1371/journal.pmed.1001275)
Supplement: Text S3 — Embase search strategy. (DOCX) [file pmed.1001275.s014.docx]

**Text S3: Embase Search Strategy**

1. exp case control study/

2. cohort analysis/

3. case control.tw.

4. (cohort adj (study or studies)).tw.

5. cohort analy$.tw.

6. (Follow up adj (study or studies)).tw.

7. (observational adj (study or studies)).tw.

8. longitudinal study/

9. retrospective study/

10. incidence/

11. (epidemiologic$ adj (study or studies)).tw.

12. 1 or 2 or 3 or 4 or 5 or 6 or 7 or 8 or 9 or 10 or 11

13. exp vein thrombosis/

14. exp venous thromboembolism/

15. exp thrombosis/

16. exp lung embolism/

17. (dvt$ or (deep$ adj8 (vein$ or ven$) adj8 thromb$) or embol$).mp.

18. 13 or 14 or 15 or 16 or 17

19. carcinoma/

20. malig$.tw.

21. neoplas$.tw.

22. oncol$.tw.

23. tumo?r$.tw.

24. neoplasm/

25. exp brain tumor/

26. exp bone tumor/

27. exp pancreas tumor/

28. exp lung tumor/

29. exp colorectal tumor/

30. exp breast tumor/

31. exp prostate tumor/

32. exp blood disease/

33. 19 or 20 or 21 or 22 or 23 or 24 or 25 or 26 or 27 or 28 or 29 or 30 or 31 or 32

34. 12 and 18 and 33

35. limit 34 to human
